# Metabolomics analysis reveals changes related to pseudocyst formation induced by iron depletion in *Trichomonas vaginalis*

**DOI:** 10.1186/s13071-023-05842-w

**Published:** 2023-07-06

**Authors:** Wei-Hung Cheng, Po-Jung Huang, Chi-Ching Lee, Yuan-Ming Yeh, Seow-Chin Ong, Rose Lin, Fu-Man Ku, Cheng-Hsun Chiu, Petrus Tang

**Affiliations:** 1 Department of Microbiology and Immunology, College of Medicine, National Cheng Kung University, Tainan, Taiwan; 2 Department of Parasitology, College of Medicine, National Cheng Kung University, Tainan, Taiwan; 3 Department of Biomedical Sciences, College of Medicine, Chang Gung University, Guishan Dist., Taoyuan City, Taiwan; 4 Genomic Medicine Core Laboratory, Chang Gung Memorial Hospital, Linkou, Taiwan; 5 Department of Computer Science and Information Engineering, College of Engineering, Chang Gung University, Guishan Dist., Taoyuan City, Taiwan; 6 Graduate Institute of Health Industry Technology, Chang Gung University of Science and Technology, Taoyuan, Taiwan; 7 Graduate Institute of Biomedical Sciences, College of Medicine, Chang Gung University, Taoyuan, Taiwan; 8 Department of Parasitology, College of Medicine, Chang Gung University, Guishan Dist., Taoyuan City, Taiwan; 9 Molecular Infectious Disease Research Center, Chang Gung Memorial Hospital, Linkou, Taiwan

**Keywords:** Amino acids, Ammonia, Cellulose, Dipeptides, Fatty acids, Glycogen, Iron deficiency, Metabolomics analysis, *Trichomonas vaginalis*

## Abstract

**Background:**

Iron is an essential element for cellular functions, such as energy metabolism. *Trichomonas vaginalis*, a human urogenital tract pathogen, is capable of surviving in the environment without sufficient iron supplementation. Pseudocysts (cyst-like structures) are an environmentally tolerated stage of this parasite while encountering undesired conditions, including iron deficiency. We previously demonstrated that iron deficiency induces more active glycolysis but a drastic downregulation of hydrogenosomal energy metabolic enzymes. Therefore, the metabolic direction of the end product of glycolysis is still controversial.

**Methods:**

In the present work, we conducted an LC‒MS-based metabolomics analysis to obtain accurate insights into the enzymatic events of *T. vaginalis* under iron-depleted (ID) conditions.

**Results:**

First, we showed the possible digestion of glycogen, cellulose polymerization, and accumulation of raffinose family oligosaccharides (RFOs). Second, a medium-chain fatty acid (MCFA), capric acid, was elevated, whereas most detected C18 fatty acids were reduced significantly. Third, amino acids were mostly reduced, especially alanine, glutamate, and serine. Thirty-three dipeptides showed significant accumulation in ID cells, which was probably associated with the decrease in amino acids. Our results indicated that glycogen was metabolized as the carbon source, and the structural component cellulose was synthesized at same time. The decrease in C18 fatty acids implied possible incorporation in the membranous compartment for pseudocyst formation. The decrease in amino acids accompanied by an increase in dipeptides implied incomplete proteolysis. These enzymatic reactions (alanine dehydrogenase, glutamate dehydrogenase, and threonine dehydratase) were likely involved in ammonia release.

**Conclusion:**

These findings highlighted the possible glycogen utilization, cellulose biosynthesis, and fatty acid incorporation in pseudocyst formation as well as NO precursor ammonia production induced by iron-depleted stress.

**Graphical Abstract:**

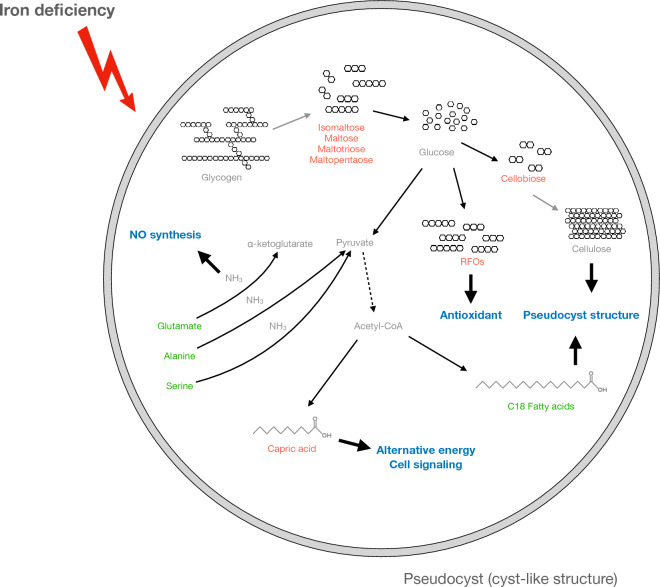

**Supplementary Information:**

The online version contains supplementary material available at 10.1186/s13071-023-05842-w.

## Background

*Trichomonas vaginalis* is the causative agent of the most common nonviral sexually transmitted disease, trichomoniasis. The high prevalence of trichomoniasis is thought to be underestimated since there are many asymptomatic infections. Symptoms of trichomoniasis range from mild inflammation to severe outcomes, such as premature labor and miscarriage [1, 2]. This infection is usually self-limited; if necessary, tinidazole and metronidazole are the first choice of treatment. However, cumulative resistance to these drugs is prompting us to develop new therapeutic strategies [3].

*T. vaginalis* resides in the urogenital tract in both sexes, where nutrient and essential elements are not supplemented sufficiently. Iron plays important biological roles in almost all living organisms. Previous reports indicated that the survival of trichomonad cells relies on different metabolic strategies in iron-deficient (ID) environments. For instance, the more active glycolysis is believed to overcome the energy-producing defect in the hydrogenosome [4, 5]. It is still controversial whether the metabolic direction of the end-product of glycolysis, pyruvate, is affected by these conditions since the downstream enzymes in the hydrogenosome are almost absent upon iron shortage [4]. Pyruvate is a central metabolite that can be converted into amino acids, lactate, and acetyl-CoA for further ATP and fatty acid biosynthesis [6]. Previous investigations indicated that *T. vaginalis* lacks a complete mechanism to digest or synthesize fatty acids de novo [7]. Consequently, less attention has been given to the regulation of lipids and related metabolites in this protist.

Cysts are the infectious stage of many human pathogenic protozoans, such as *Entamoeba histolytica* and *Giardia intestinalis*. The metabolism in cyst-formed cells is different than that in the active trophozoite. During the *E. histolytica* encystation, glycogen and lipid metabolism are more active for cyst wall formation and membranous rearrangement, respectively [8–11]. Similar metabolic events are also present in *G. lamblia*, although the major component of the cyst wall is β-1,3-GalNac rather than chitin in *E. histolytica* [12].

Iron deficiency and other environmental challenges, such as cold temperature, induce the morphological transformation of *T. vaginalis* from trophozoite to pseudocyst (cyst-like structure) [13, 14]. Unlike *E. histolytica* and *G. lamblia*, *T. vaginalis* does not undergo an encystation process and lacks a cyst wall. We had known that glycogen accumulated and was consumed rapidly in *T. vaginalis* during the logarithmic phases in axenic culture [15]. An increase in chitin-/cellulose-based content has been shown in the pseudocyst stage [14]. However, either the utilization of glycogen or chitin/cellulose biosynthesis regulated by iron shortage in *T. vaginalis* is still largely unknown.

*T. vaginalis* utilizes simple and complex saccharides for its proliferation. Trussell and Johnson demonstrated that the protist does not use cellobiose, sucrose, or raffinose as the energy source [16]. However, a recent report suggested that trichomonad cells encode a β-fructosidase that is capable of degrading sucrose and raffinose [17]. Raffinose family oligosaccharides (RFOs) are mainly found in plants and are generated as storage/transport sugars. Additionally, RFOs also participate in distinct cellular processes, such as antioxidant activity and signal transduction [18]. Nonetheless, the role of RFOs has not been addressed in *T. vaginalis*.

In this study, we aimed to illustrate the compositional changes in metabolites in *T. vaginalis* induced by iron shortage. By using LC‒MS-based metabolomics analysis, the pyruvate-centered metabolic directions were highlighted, and pseudocyst-associated compounds were also discussed. We found possible glycogen breakdown, cellulose biosynthesis, and RFO accumulation in ID cells. Most fatty acids were comparable in the ID and iron-rich (IR) controls, with the expectation of a significant elevation of capric acid. C18 fatty acids were mostly decreased, which implied their incorporation into membranous structures during pseudocyst formation. Amino acids were almost reduced, especially alanine, glutamate, and serine, which could be explained by the accumulation of dipeptides. The enzymatic reactions in the opposite direction of alanine, glutamate, and serine were accompanied by ammonia release, which might be a crucial substrate for nitric oxide (NO) production. Collectively, this untargeted metabolomics analysis showed that saccharides, fatty acids, and amino acids were functionally associated with pseudocyst formation in *T. vaginalis* induced by iron limitation. These findings provide new insights into the unique mechanisms during infection and provide potential targets for further investigations.

## Methods

### Cell culture and treatments

*Trichomonas vaginalis* (ATCC_30236) was cultured in YIS medium containing iron (ferric ammonia citrate, 180 µM) or the iron chelator dipyridyl (180 µM) as the iron-rich (IR) and iron-deficient (ID) groups, respectively [23]. IR cells were collected when they reached a density of ~ 2 × 10^6^/ml, whereas ID cells were collected at 6 h after dipyridyl (DIP, iron chelator) treatment [5].

### Metabolite extraction

For each sample, 1000 μl of extract solution (acetonitrile:methanol:water = 2:2:1) containing internal standard (L-2-chlorophenylalanine, 2 μg/ml) was added. After 30 s of vortexing, the samples were homogenized at 30 Hz for 4 min and sonicated for 5 min in an ice-water bath. The homogenization and sonication cycle was repeated twice. Then, the samples were incubated at − 40 °C for 1 h and centrifuged at 10,000 rpm for 15 min at 4 °C. A total of 750 μl supernatant was transferred to a fresh tube and dried in a vacuum concentrator at 37 °C. Then, the dried samples were reconstituted in 200 μl of 50% acetonitrile by sonication on ice for 10 min. The constitution was then centrifuged at 12,000 rpm for 15 min at 4 °C, and 75 μl of supernatant was transferred to a fresh 2 ml LC–MS glass vial.

### LC‒MS/MS analysis

UHPLC separation was carried out using a 1290 Infinity series UHPLC System (Agilent Technologies) equipped with a UPLC BEH Amide column (2.1 × 100 mm, 1.7 μm, Waters). The mobile phase consisted of 25 mmol/l ammonium acetate and 25 ammonia hydroxide in water (pH = 9.75) (A) and acetonitrile (B). The analysis was carried out with an elution gradient as follows: 0–0.5 min, 95% B; 0.5–7.0 min, 95–65% B; 7.0–8.0 min, 65–40% B; 8.0–9.0 min, 40% B; 9.0–9.1 min, 40–95% B; 9.1–12.0 min, 95% B. The column temperature was 25 °C. The autosampler temperature was 4 °C, and the injection volume was 1 μl (pos) or 1 μl (neg).

TripleTOF 6600 mass spectrometry (AB Sciex) was used to acquire MS/MS spectra on an information-dependent basis (IDA) during an LC–MS experiment. In this mode, acquisition software (Analyst TF 1.7, AB Sciex) continuously evaluated the full scan survey MS data as it collected and triggered the acquisition of MS/MS spectra depending on preselected criteria. In each cycle, the 12 most intensive precursor ions with intensities. 100 were chosen for MS/MS at a collision energy (CE) of 30 eV. The cycle time was 0.56 s. ESI source conditions were set as follows: gas 1, 60 psi; gas 2, 60 psi; curtain gas, 35 psi; source temperature, 600 °C; declustering potential, 60 V; ion spray voltage floating (ISVF), 5000 V and − 4000 V in positive or negative modes, respectively [19].

### Data preparation and annotation

MS raw data (.wiff) files generated from the previous steps were converted to the mzXML format by ProteoWizard and processed by R package XCMS (version 3.2). The process included peak deconvolution, alignment, and integration. Minfrac and cutoff were set as 0.5 and 0.6, respectively. An in-house MS2 database was applied for metabolite identification [20].

### Statistical analysis

The reproductivities of two metabolomics sets were analyzed by orthogonal partial least-squares discriminant analysis (OPLS-DA). Student’s t-test and variable importance in the projection (VIP) were performed to highlight significant changes in metabolites. Asterisks were used to represent the significance of each assay as determined through the *P* value (*P < 0.05; **P < 0.01; ***P < 0.001).

## Results and discussion

### Metabolomics analysis of iron-rich and iron-deficient cultured *T. vaginalis*

**Fig. 1:**
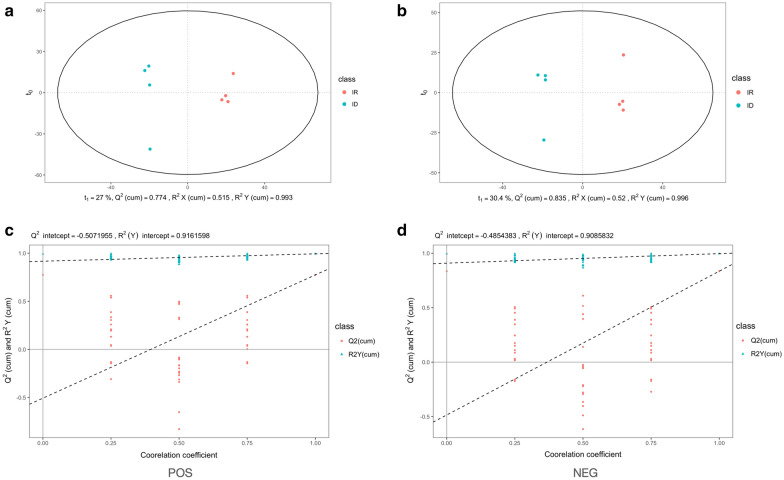
Multivariate analysis of the UHPLC-MS data from iron-rich (red) and iron-deficient (blue) groups. OPLS-DA score plots (**a**, **b**) and permutation analysis (**c**, **d**) (permutation test *n* = 999) of positive and negative ion modes

Four equal samples from IR and ID trichomonad cells were collected for HPLC-QTOF MS analysis. Generally, principal component analysis (PCA) was performed to show the correlation of the metabolomics dataset among the experimental groups. However, due to the small sample size and multiple dimensions of detected metabolites, PCA was not suitable for our experiment (data not shown). OPLS-DA is another method to measure the similarities and differences between experimental settings (IR and ID). The score plot of OPLS-DA revealed a correlation within IR and ID datasets in positive and negative ion modes (Fig. 1a, b). In addition, permutation tests (*n* = 999) for each ion mode were performed to further confirm the validity of the two experimental groups (Fig. 1c, d). The *R*^2^*Y* and *Q*^2^ values were > 0.5, suggesting that these clustering trends of major components derived from samples were separated expectedly in both positive and negative ion modes.

A total of 3277 peaks were present in positive and negative ion modes (1802 and 1475, respectively) (Table 1). Among them, 824 metabolites were annotated after MS/MS analysis (~ 25% of total compounds). The annotation rate of human metabolomics analysis is approximately 10% [21, 22], suggesting that our annotation rate was reasonable and suitable for further investigations. The annotated compounds are listed in Additional file 1.

**Table 1:** Statistics of the compounds identified in this study

	Metabolites	With annotation
Positive ion	1802	536
Negative ion	1475	288
Total	3277	824

**Fig. 2:**
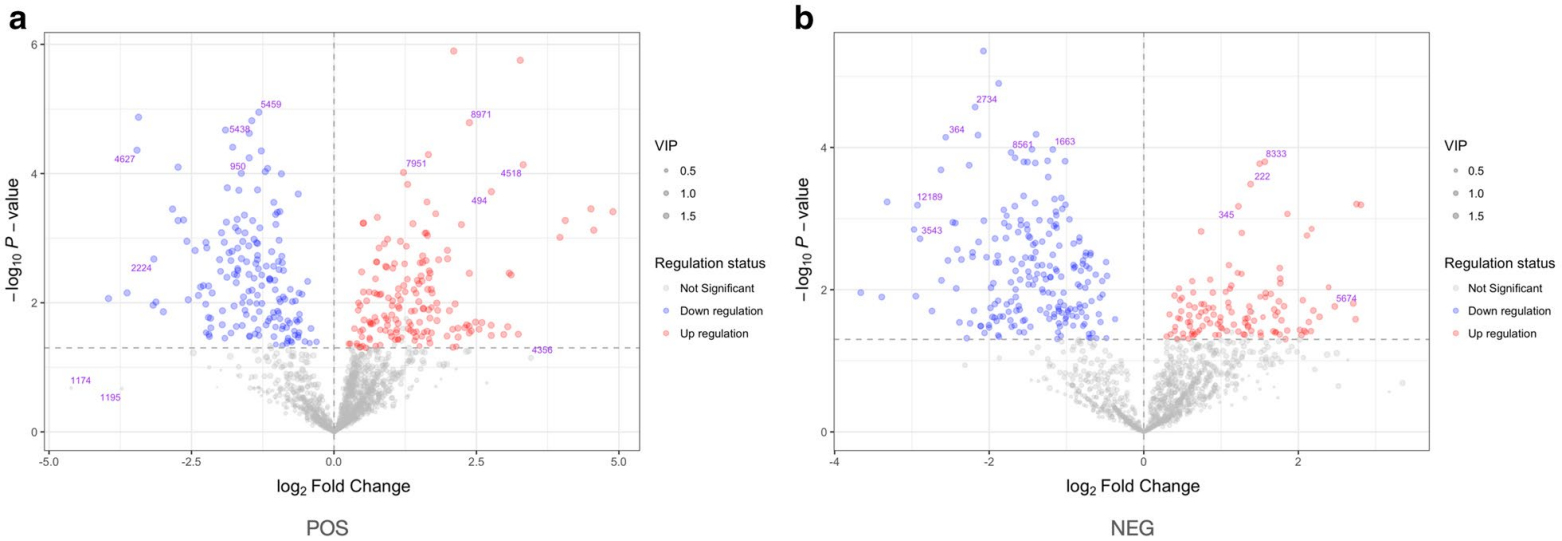
Volcano plots of significantly changed compounds in positive (**a**) and negative (**b**) ion models. Red dots represent compounds upregulated significantly in IR cells. Blue dots represent compounds downregulated significantly in IR cells. VIP score values are indicated by the size of dots. IR, iron-rich; ID, iron-deficient

There were 667 compounds derived from positive and negative ion modes that exhibited significant changes between the IR and ID groups according to the volcano plots and VIP values (Fig. 2). Blue dots represent the upregulated compounds, whereas red dots indicate the downregulated compounds in IR cells compared to ID cells. The size of the dots indicates the VIP scores.

Our previous report suggested that glycolysis is highly active in ID *T. vaginalis* [23]. However, pyruvate would not be converted into acetyl-CoA for ATP and fatty acid production because of the global downregulation of genes/proteins inside hydrogenosomes under ID conditions [4, 5]. To better understand the fate of pyruvate upon iron depletion, we concentrated on pyruvate-related compounds in this study.

### Saccharide metabolism

**Fig. 3:**
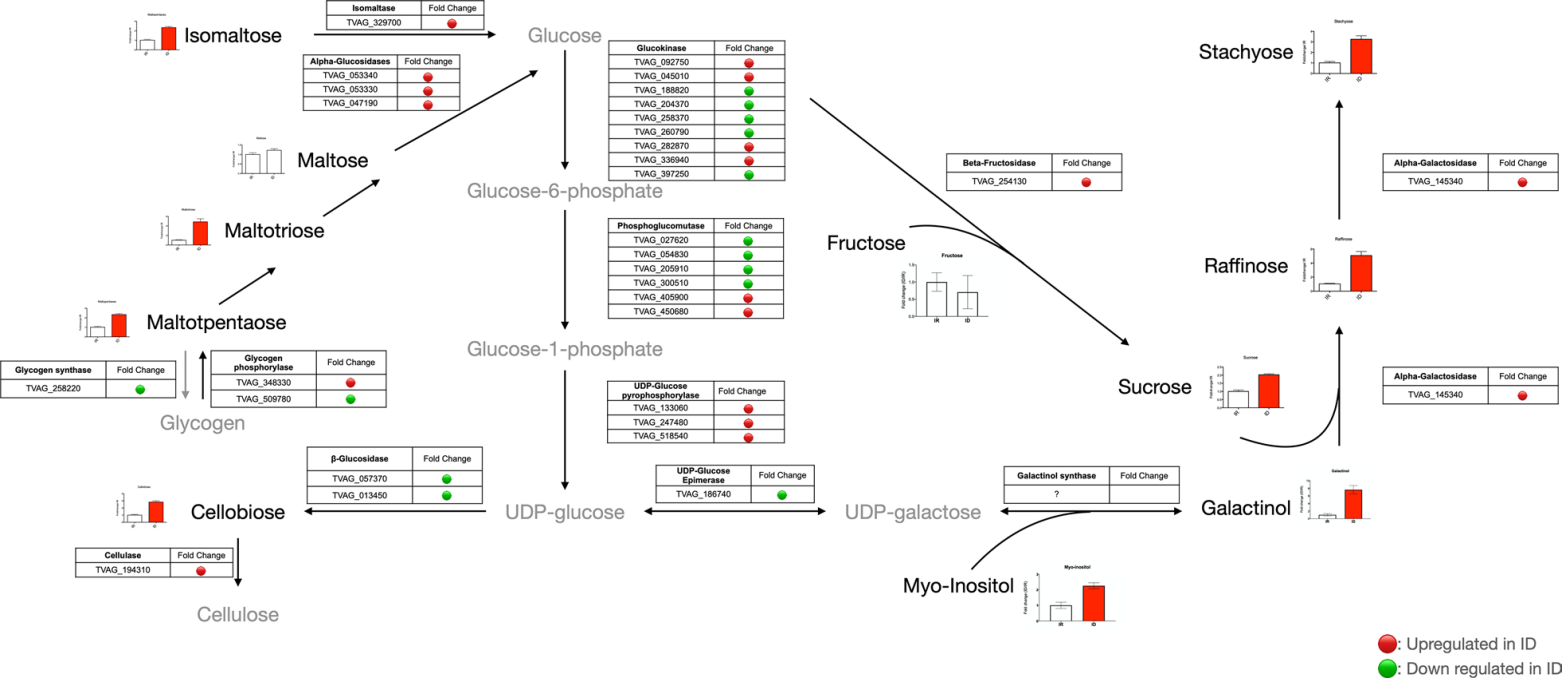
Oligosaccharide metabolism in *Trichomonas vaginalis* cultured under different iron concentrations. The levels of oligosaccharides detected in this analysis are shown in bar charts. Significantly regulated compounds are shown in red (*P* < 0.05). The corresponding enzymes are listed by the arrows, and relative RNA expression levels (ID/IR) are indicated. Genes upregulated in ID cells are shown in red, while genes downregulated in ID cells are shown in green [5]. The gray compounds represented not being detectable in this analysis. Galactinol synthase is lacking in *T. vaginalis*

The first group of compounds that we focused on were oligosaccharides since they were altered significantly in ID cells. Figure 3 highlights the most accumulated oligosaccharides in ID cells compared to that of the IR control.

#### Accumulation of ɑ-1,4-linked oligosaccharides in ID cells

The left portion of Fig. 3 shows that isomaltose, maltotriose, and maltopentaose increased significantly, except maltose. These di-, tri-, and penta-saccharides were generated from either glucose polymerization or glycogen degradation. The expressions of enzymes related to these reactions, isomaltase and ɑ-glucosidases, were increased in ID cells (Fig. 3). The expression of one glycogen phosphorylase (TVAG_348330) was slightly upregulated, whereas glycogen synthase was downregulated under iron-limited conditions (Fig. 3). According to the expression profiles of glycogen metabolic enzymes, we speculated that the direction of glycogen and oligosaccharide metabolism was toward degradation.

Glycogen is a storage saccharide reported to accumulate and be consumed rapidly in the log phase of axenic cultured *T. vaginalis*. The activity of glycogen phosphorylase exhibited the same trend as the glycogen level in the previous measurement [15]. Glycogen consumption along with more active glycolysis during the encystation process has been demonstrated in *E. histolytica* and *G. lamblia* [24–26]. In the case of iron shortage, the transition to pseudocysts and more active glycolysis implied that *T. vaginalis* underwent an encystation-like event similar to those intestinal protozoa. The possible utilization of glycogen is not only for energy metabolism but also provides a substrate for cyst wall synthesis, such as chitin [25]. The true cyst wall is absent in the pseudocyst of *T. vaginalis*; however, it would be a catalytic direction from glucose to cellulose. This will be discussed in the following section.

#### Cellobiose accumulation in ID cells

Another disaccharide was highlighted because of the drastic elevation in ID cells, cellobiose, compared to the IR control (Fig. 3). Cellobiose is the building block of cellulose, a structural polymer for plants and cysts [12]. The enzyme responsible for the conversion between NDP-glucose and cellobiose, β-glucosidase, was downregulated in ID cells. Conversely, the upregulation of cellulase (TVAG_194310), which converts cellulose to cellobiose, was found in ID cells. Notably, cellulase catalyzes bidirectional reactions, which are also responsible for cellulose biosynthesis [27]. The evidence might imply that cellulase is involved in cellulose biosynthesis rather than breakdown to cellobiose.

The interconversion of cellobiose and polymeric cellulose is functionally associated with cell structure. Previous investigations demonstrated that polysaccharides, including chitin, acid-fast lipids, and cellulose, are the main backbone for cyst wall formation in distinct encysting protozoa [12]. Pseudocysts of *T. vaginalis* are built up with a chitin-/cellulose-based architecture [14]. Although there are four chitinase genes encoded by *T. vaginalis*, none of them have been evaluated. Based on our RNA expression dataset, all chitinases were not significantly altered between IR and ID conditions [5]. We first demonstrated the presence of cellobiose, a disaccharide with a β-1,4-glycosidic linkage of glucoses, which is also the substrate for cellulose biosynthesis. The accumulation of cellobiose was accompanied by downregulation of β-glycosidase and upregulation of cellulase in ID cells. Moreover, cellobiose is not used for the proliferation of trichomonad cells [16]. Collectively, we speculated that cellulose biosynthesis might be involved in the progression of ID cells during pseudocyst formation.

#### Raffinose family oligosaccharides (RFOs) accumulated in ID cells

We have stated above that glycogen storage and structural cellulose metabolism are affected by iron depletion. Surprisingly, our dataset showed that raffinose family oligosaccharides (RFOs), including sucrose, raffinose, and stachyose, also significantly accumulated under ID conditions (Fig. 3). RFOs are saccharides composed of fructose, glucose, and galactose. In the synthetic pathway, galactinol is first generated from UDP-galactose and myo-inositol by galactinol synthase. Galactinol and sucrose are substrates for raffinose synthesis catalyzed by ɑ-galactosidase. Stachyose is subsequently generated via the combination of raffinose and galactinol [28]. Levels of myo-inositol and galactinol were dramatically elevated in ID cells (Fig. 3, bottom right). The RNA levels of enzymes related to RFO synthesis, β-fructosidase (TVAG_254130), and ɑ-galactosidase (TVAG_145340) were also upregulated in ID cells. Although UDP-galactose was not detected and galactinol synthase was absent in *T. vaginalis*, evident elevations in galactinol and the downstream RFOs led us to hypothesize that trichomonad cells produced different forms of sugars to respond to unfavorable environments.

RFOs are broadly distributed in the plant kingdom and are especially well studied in soybean and chickpea [18]. These oligosaccharides participate in several biological functions, such as carbon storage and transport, tolerance to environmental stresses, and human gut microbiome manipulation [18]. A previous investigation demonstrated that recombinant trichomonad β-fructosidase is capable of degrading sucrose and raffinose [17]. However, as a storage sugar, raffinose is not utilized for the growth of *T. vaginalis* [29]. These statements suggested that raffinose, sucrose, and stachyose were not likely to be used for energy production and cell proliferation.

RFOs accumulate under iron-depleted conditions, and they act as stress-responsive molecules in plants [30, 31]. The function of accumulated RFOs reflects the involvement of environmental changes in cases of changes in osmolarity, temperature, and infections [32]. Furthermore, RFOs possess hydroxyl radical scavenging activity that would be an important antioxidant [33]. We suggested that reactive nitrogen species (RNS) rather than reactive oxygen species (ROS) accumulated in ID trichomonad cells. The imbalance of redox homeostasis in iron-limited circumstances induces the thioredoxin-peroxiredoxin system in *T. vaginalis* [5]. Accordingly, we speculated that the increase in RFOs might also be an antioxidant induced by iron shortage.

Collectively, we hypothesized that glycogen was used as the resource for more active glycolysis; on the other hand, cellulose biosynthesis occurred at the same time for pseudocyst formation. RFO accumulation revealed a possible antioxidative or signaling role in response to iron shortage, which should be examined in detail in the future.

### Lipid metabolism

Previous studies have demonstrated that *T. vaginalis* possesses defective machinery to synthesize lipid-related biomolecules, such as fatty acids [7]. The draft genome also showed that no complete fatty acid beta-oxidation or biosynthesis genes are encoded by the protist [34]. However, we identified different kinds of lipids in the metabolomics analysis.

#### Reduction in C18 fatty acids levels in ID cells

**Fig. 4:**
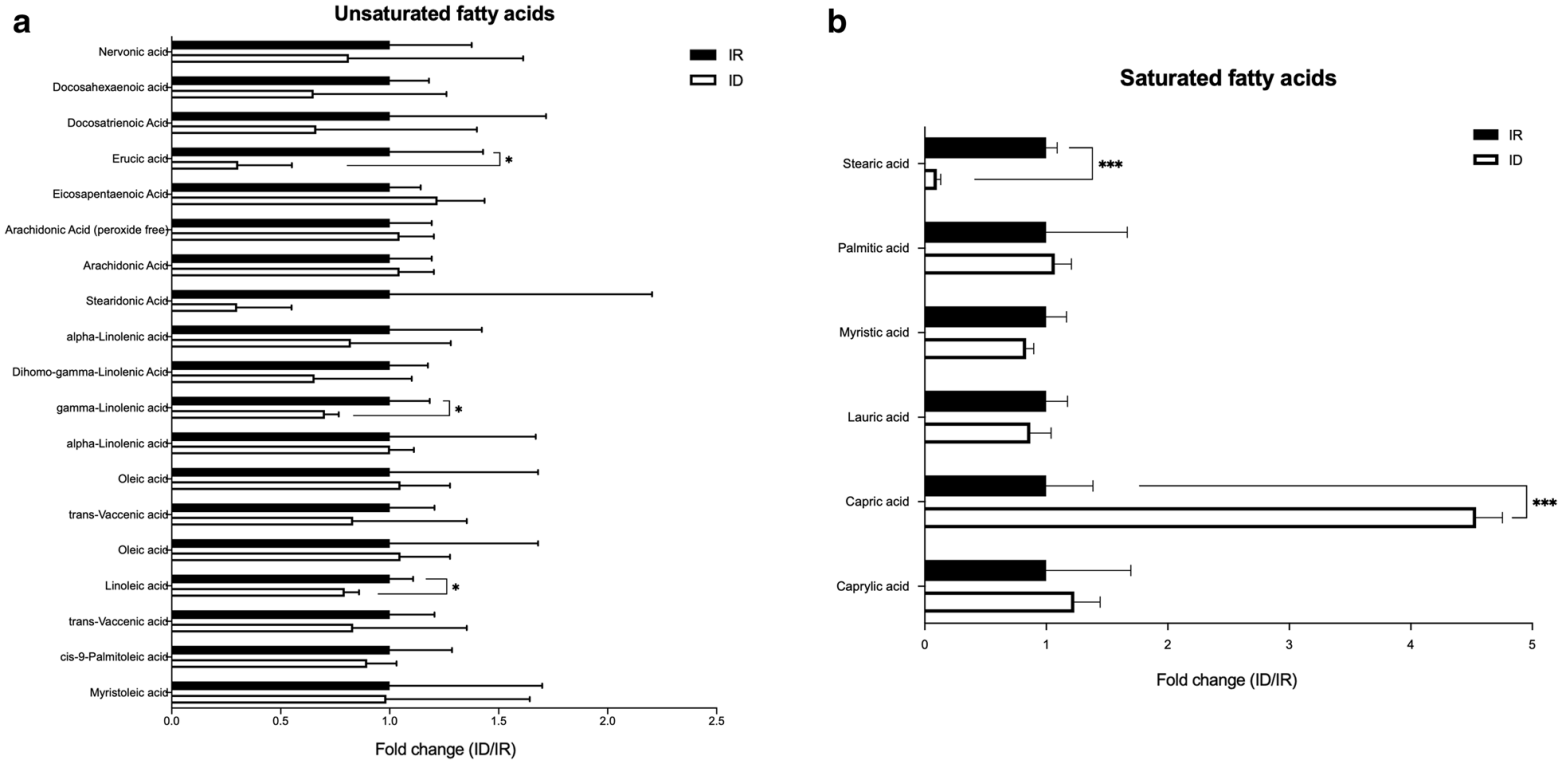
Levels of unsaturated (**a**) and saturated (**b**) fatty acids identified in the metabolomics analysis. The relative values of fatty acids identified are shown as the fold change from ID (open bar) to IR (closed bar) (ID/IR) cells. Significance is indicated by asterisks: *P < 0.05; **P < 0.01; ***P < 0.001. IR, iron-rich; ID, iron-deficient

Figure 4 shows that unsaturated fatty acid levels were mostly comparable, except for significant reductions in the contents of linoleic acid (C18), gamma-linolenic acid (C18), and erucic acid (C22) in ID cells. Additionally, a drastic decrease in the stearic acid (C18) content in ID cells was shown in the category of saturated fatty acids. These results indicated a greater utilization or lower synthesis of multiple C18 fatty acids in ID trichomonad cells.

Alterations in lipid metabolism might be involved in the morphological changes in human pathogenic protozoa, such as encystation. In *G. lamblia*, the composition of fatty acids has been investigated, and it was found that stearic (C18) and oleic (C18) acids are the most abundant fatty acids in *Giardia* during encystation [35]. *Trichomonas vaginalis* underwent a transformation from motile trophozoites to arrested pseudocysts under iron-limited conditions [13]. The composition of fatty acids in ID trichomonad cells (or pseudocysts) revealed a global decrease in C18 fatty acids (Fig. 4). The difference between previous and present studies is the stage of protists: encysting *G. lamblia* and pseudocysts of *T. vaginalis*. Moreover, lipid composition regulates the encystation process of *E. histolytica* [11, 36]. Hence, we hypothesized that these C18 fatty acids were likely incorporated into membranous structures during the progression of encystation.

Although transacylation activity participates in the generation of several lipids, previous studies revealed that *T. vaginalis* is unable to synthesize fatty acids [37]. However, medium-derived fatty acids [mainly medium-chain fatty acids (MCFAs)] are incorporated into phosphoglyceride and sphingolipids [7]. Indeed, the detectable phospholipids in our results were significantly reduced, especially derivatives of phosphoethanolamides (PEs) (Additional file 2). Derivatives of phosphatidylcholine, choline, and serine were not significantly changed in the metabolomics analysis (data not shown). Reductions in the detected fatty acids and phospholipids were likely incorporated into the membranous structure in *T. vaginalis*. Collectively, these results implied that iron shortage lowered most detected C18 fatty acids via putative fatty acid incorporation into phospholipids, which might be associated with pseudocyst formation in *T. vaginalis*.

#### Accumulation of capric acid in ID cells

Due to the lack of related enzymes in *T. vaginalis*, the composition of fatty acids had not been documented in detail previously. A gene annotated as 2-nitropropane dioxygenase precursor (TVAG_479680), also known as enoyl-acyl carrier protein reductase (ENR), participates in fatty acid biosynthesis [38]. ENR catalyzes the final step of fatty acid elongation of the type II fatty acid synthesis (FAS II). The expression of TvENR showed a significant elevation under ID conditions [5]. Here, we showed that a putative ENR-dependent synthesized capric acid was elevated under ID conditions, while most of them were comparable except stearic acid (discussed above) (Fig. 4b). This result suggested that a functional FAS II would exist in *T. vaginalis*, and the downstream fatty acid could be changed by iron depletion.

Acetyl-CoA is the essential building block of fatty acids, which was thought to be reduced in ID cells since the enzyme pyruvate:ferredoxin oxidoreductases (PFOs) decreased drastically in ID cells [5, 34]. We suggest that the accumulation of capric acid might utilize acetyl-CoA originating from neither glucose nor the remaining known substrates or generated from the degradation of longer fatty acids, such as stearic acid (C18) [37]. Nonetheless, whether the transportation of fatty acids from the culture medium was induced by iron limitation could still not be ruled out.

Lipids are an important energy source, especially with limited sugar supplementation. Previous investigations revealed that enzymatic activities associated with β-oxidation are absent in *T. vaginalis* [37]. Here, we showed that at least three unsaturated fatty acids were significantly reduced in ID cells (Fig. 4). The mRNA expression of long-chain fatty acid acyl-CoA synthetase, the first enzymatic step in the oxidation of fatty acids, increased slightly, further implying the usage of fatty acids by *T. vaginalis* [5]. However, this hypothesis should be verified in the future.

The only accumulated fatty acid identified in this study is capric acid (known as decanoic acid), an MCFA. MCFAs (composed of 7–12 carbons) have important biological functions in cells [39]. Previous reports demonstrated that MCFAs are crucial energy storage molecules. The relatively short carbon chain is an advantage for transportation into mitochondria and is therefore easier to catalyze [40]. Furthermore, fewer ROS are generated during MCFAs β-oxidation compared to long-chain fatty acids [41]. In addition to energy production, regulatory roles of MCFAs have been revealed, such as the enhancement of glycolysis in the brain [42]. This phenomenon reflects the fact that glycolysis is more active in *T. vaginalis* upon iron depletion [23]. Gluconeogenesis is also enhanced by MCFAs. However, the key enzymes are absent in *T. vaginalis* [34, 43]. Therefore, the accumulation of capric acid might be linked to the metabolic regulation of *T. vaginalis* under iron shortage.

Taken together, our results suggested that the utilization of C18 fatty acids was in response to the transformation of pseudocysts. Energy metabolism, such as glycolysis and fatty acid catalysis, was altered by iron depletion, which was possibly regulated by the significant accumulation of capric acid.

### Amino acid metabolism

Catabolism of many amino acids relies on the presence of pyruvate. We demonstrated more active proteolysis via the ubiquitin‒proteasome system (UPS) triggered by iron shortage [5]. Moreover, several proteolytic enzymes were upregulated in ID cells. Proteolysis is responsible for the clearance and recycling of amino acids. We consequently assumed that amino acids would accumulate in trichomonad cells under iron-limited conditions. However, the dataset showed a decrease in amino acid levels in ID cells.

#### Alanine, glutamate, and serine were significantly reduced in ID cells

**Fig. 5:**
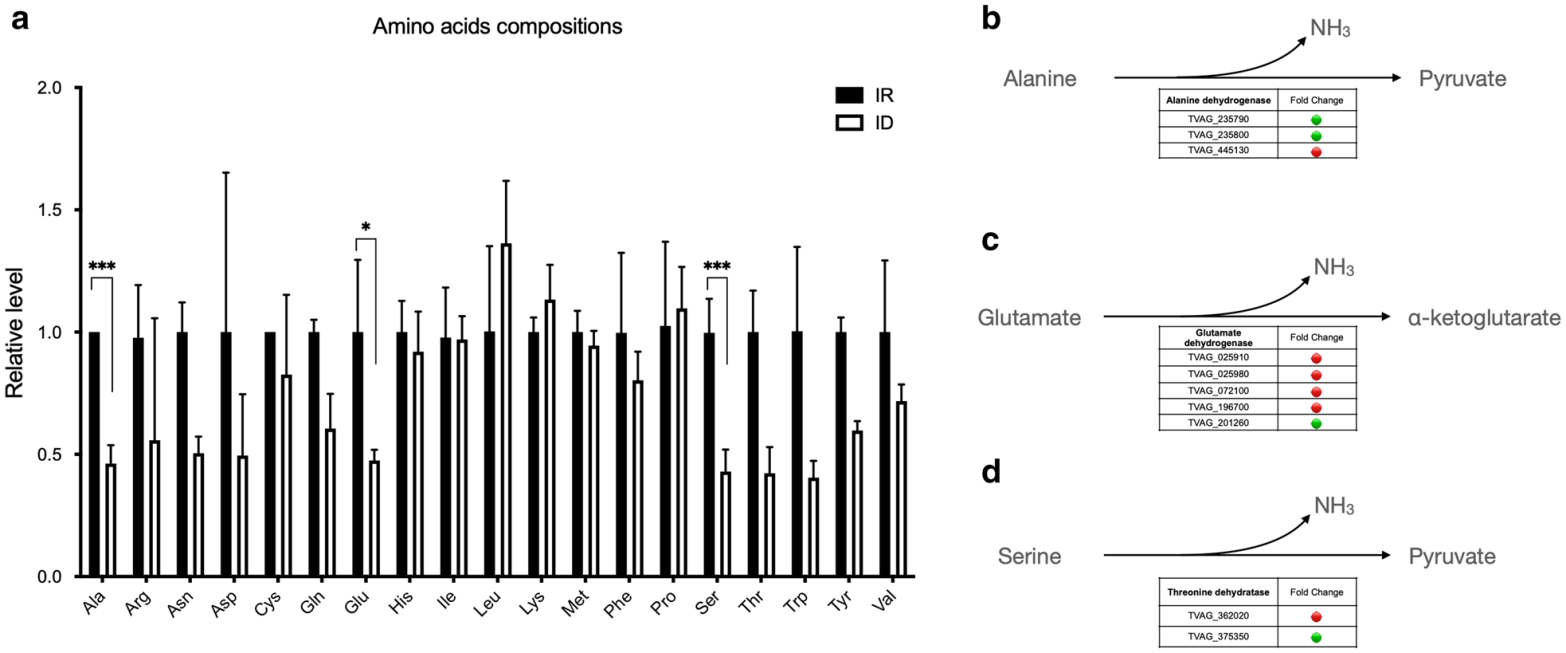
Amino acid composition in *T. vaginalis* cultured in different iron concentrations. Amino acids detected in IR (closed bars) and ID (open bars) are shown (**a**). Significance is indicated by asterisks: *P < 0.05; **P < 0.01; ***P < 0.001. **b**–**d** Reactions of alanine dehydrogenase, glutamate dehydrogenase, and threonine dehydratase are shown. Relative RNA expression levels (ID/IR) of genes are indicated in red (upregulated in ID) and green (downregulated in ID) colors [5]. IR, iron-rich; ID, iron-deficient

Among the detected amino acids, only alanine, glutamate, and serine were significantly reduced in ID cells (Fig. 5a). Alanine dehydrogenase and threonine dehydratase catalyze the conversion of alanine and serine to pyruvate, respectively. In addition, glutamate dehydrogenase is responsible for the conversion between glutamate and ɑ-ketoglutarate (Fig. 5b–d). It is noticeable that all these reactions were accompanied by the release of ammonia. Other amino acid catabolisms associated with ammonia release, including arginine, asparagine, cysteine, glutamine, methionine, threonine, and tryptophan, were all reduced compared to the IR control, although without significance (Fig. 5a). Therefore, these amino acids were reduced with a similar upregulation trend as the corresponding enzymes in ID cells, implying that these reactions catalyzed in the opposite direction that generated ammonia.

Glutamate is the metabolic center for several amino acids [34]. Leucine and lysine showed unchanged or slightly elevated levels in ID cells. The enzyme catalyzes the conversion of glutamate to isoleucine, leucine, and valine, branched-chain amino acid (BCAA) aminotransferases (TVAG_026740), which are upregulated under ID conditions, while lysine aminotransferase is undetectable at the RNA level [5]. The slight increase in leucine and lysine implied that the catalytic directions of glutamate were not only toward ɑ-ketoglutarate but also toward leucine and lysine (Fig. 5a).

Nitric oxide (NO) plays a crucial survival role in ID trichomonad cells; however, arginine-dependent NO generation occupied only ~ 20% of the total NO, indicating that most NO was synthesized via different unknown machineries [5]. Ammonia is a nitrogen-containing compound that can be converted to NO by oxidation [44, 45]. Ammonia metabolism takes place in the hydrogenosome of *T. vaginalis* by the activity of hydroxylamine reductase (HCP, TVAG_336320), which is highly expressed in ID cells [46]. Additionally, HCP is known to scavenge NO in the bacterial model, indicating a possible linkage between ammonia and NO metabolism in *T. vaginalis* [47]. Therefore, we hypothesized that ammonia derived from amino acid catabolism might be an important substrate for NO production in *T. vaginalis* under iron-depleted conditions.

#### Dipeptides significantly accumulated in ID cells

**Fig. 6:**
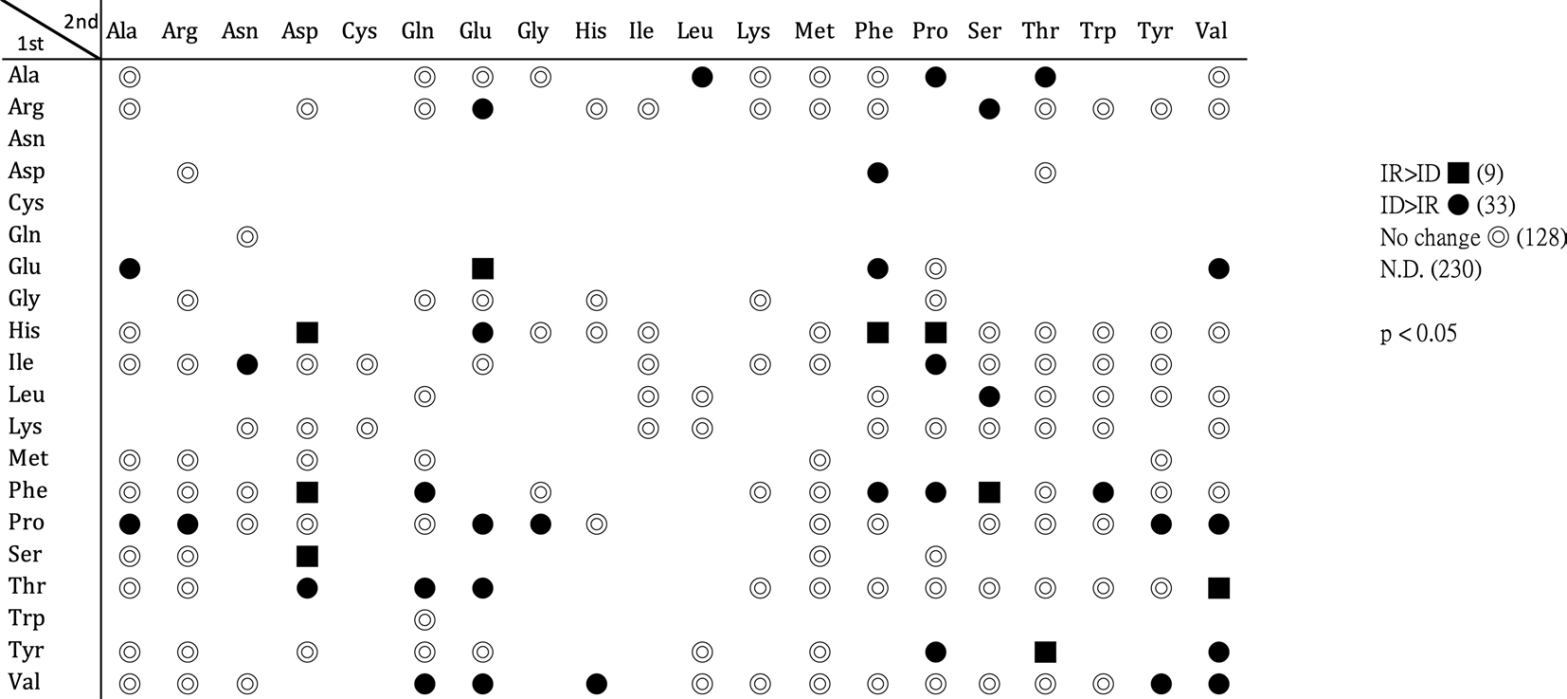
Alternations in dipeptide amounts in *T. vaginalis* cultured in different iron concentrations. The relative levels of identified dipeptides are listed in this chart. A total of 170 dipeptides were identified in this work. Dipeptides without significant differences are labeled with ◎. Dipeptides accumulated in IR cells are labeled with black-filled square, while dipeptides accumulated in ID cells are labeled with black-filled circle (*P* < 0.05)

Most amino acids reduced in ID cells have been discussed above; however, whether mechanisms other than amino acid catabolism are involved is not clear. A fraction of dipeptides occupied the annotated compounds of the dataset and attracted our attention. Dipeptides are composed of two amino acids linked with peptide bonds and are generated either proteolytically or proteinogenically [48, 49]. There are 400 possible combinations of dipeptides theoretically. In the present investigation, we identified 170 dipeptides (Fig. 6). Among them, 33 accumulated in ID cells, whereas 9 were elevated in IR cells. This result could be an explanation, in part, for the overall amino acid level reduction in ID cells being caused by dipeptide accumulation. Dipeptides with N-terminal proline, valine, and phenylalanine were the most abundant in ID cells. In fact, the expressions of dipeptidases and dipeptidyl peptidases were mostly upregulated in ID cells; thus, the synthetic mechanism of dipeptides could not be determined [5].

Dipeptides composed of cysteine residues are linked to antioxidation activity [50]. As the imbalance of redox caused by iron depletion, however, the only identified cysteine-containing dipeptides, Ile-Cys and Lys-Cys, were not changed by the iron content in *T. vaginalis*. Dipeptides also play regulatory roles. The glycolytic enzyme glyceraldehyde-3-phosphate dehydrogenase (GAPDH) is the target of the Tyr-Asp dipeptide. The interaction between GAPDH and Tyr-Asp results in the restriction of enzymatic activity [51]. This suggested that these stress-specific accumulated dipeptides might participate in biological regulation in *T. vaginalis*.

In summary, the overall reduction in the levels of amino acids in ID cells might be associated with the accumulation of dipeptides. In addition, significantly reduced alanine, glutamate, and serine were accompanied by the release of ammonia, which was likely the substrate for NO production in the protist under iron-deficient environments.

## Conclusion

**Fig. 7:**
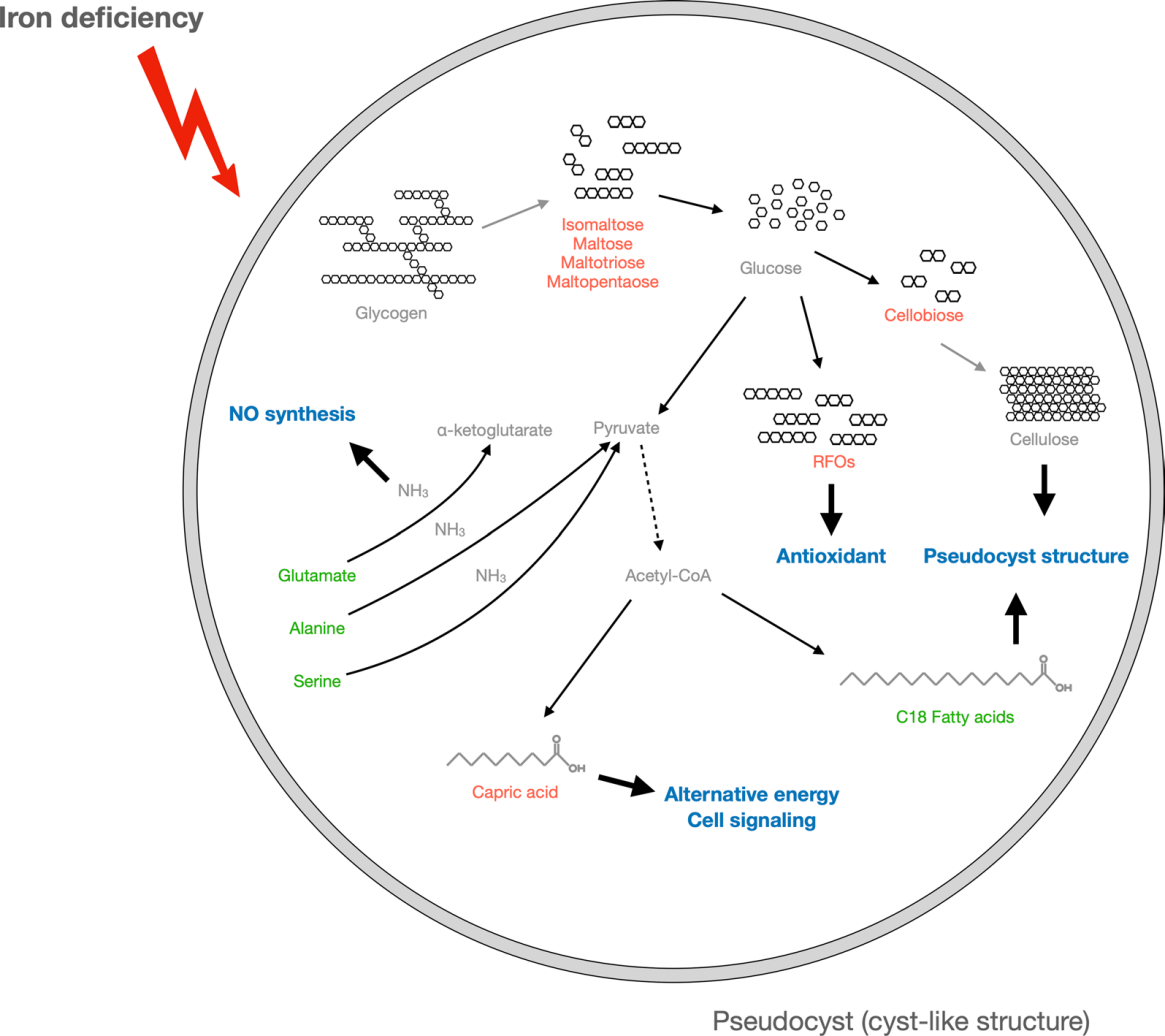
The proposed model of metabolic changes in *T. vaginalis* under iron-limited environments. Once trichomonad cells encountered iron-deficient conditions, most of the cells transformed into pseudocysts. The carbon source of the more active glycolysis was derived from glycogen hydrolysis because of the accumulation of ɑ-1,4-linked, glucose-composed oligosaccharides. The accumulation of cellobiose was a possible hint for cellulose biosynthesis, which is the crucial conformational component of pseudocysts. C18 fatty acids reduction implied incorporation into phospholipids for pseudocyst formation. Capric acid accumulation might be involved in the regulation of glycolysis or as a signaling molecule in response to iron-limited environments. Amino acid reduction likely resulted from dipeptide accumulation or incomplete proteolysis. In addition, the significant reductions in alanine, glutamate, and serine were all involved in the release of ammonia, which was likely a key resource for nitric oxide synthesis in *T. vaginalis*. Compounds labeled in red and green colors represent accumulation and reduction in ID cells, respectively. Gray-colored metabolites were the speculated metabolites associated with the detected compounds. The putative molecular function or pathways were labeled in blue color

In this study, we conducted an untargeted metabolomics analysis to illustrate the metabolic directions of glucose and pyruvate. We found that possible glycogen degradation, cellulose polymerization, and RFO synthesis occurred at the same time for more active glycolysis, pseudocyst formation, and antioxidation, respectively. Capric acid was the only fatty acid that accumulated in ID trichomonad cells, while most of the C18 fatty acids were significantly reduced. This might be due to membranous structure alterations during pseudocyst formation. Last, the overall amino acid level reduction was probably caused by dipeptide accumulation. Ammonia release from reactions of alanine, glutamate, and serine catabolism was the possible substrate for NO production in *T. vaginalis* upon iron deficiency. These findings provide information on metabolic changes mainly toward pseudocyst formation induced by iron depletion. A proposed model is shown in Fig. 7.

## Supplementary Information

**Additional file 1. File 1**: The list of annotated compounds identified in the metabolomics analysis.

**Additional file 2. File 2**: The relative amount of phospholipid derivatives identified in the metabolomics analysis. The relative values of phospholipid derivatives identified are shown as the fold-change from ID (gray bar) to IR (black bar) (ID/IR) cells. Significance is indicated by asterisks: *P < 0.05; **P < 0.01; ***P < 0.001. IR, iron-rich; ID, iron-deficient.

## Data Availability

All data generated or analyzed during this study are included in this published article (and its Additional files 1 and 2).

## Abbreviations

ID: Iron-deficient
IR: Iron-rich
RFOs: Raffinose family oligosaccharides
MCFA: Medium-chain fatty acid
NO: Nitric oxide
OPLS-DA: Orthogonal partial least-squares discriminant analysis
VIP: Variable importance in the projection
PCA: Principal component analysis
HPLC-QTOF: High performance liquid chromatography-quadrupole time of flight
RNS: Reactive nitrogen species
ROS: Reactive oxygen species
PEs: Phosphoethanolamides
ENR: Enoyl-acyl carrier protein reductase
FAS II: Type II fatty acid synthesis
PFOs: Pyruvate:ferredoxin oxidoreductase
UPS: Ubiquitin–proteasome system
BBCA: Branched-chain amino acid
HCP: Hydroxylamine reductase
GAPDH: Glyceraldehyde-3-phosphate dehydrogenase
