# Supplementary figures and images for: Metabolomics analysis reveals changes related to pseudocyst formation induced by iron depletion in Trichomonas vaginalis
Source: Parasit Vectors. 2023 Jul 6;16:226. doi: 10.1186/s13071-023-05842-w (PMC10327357; doi:10.1186/s13071-023-05842-w)

# Derivatives of phospholipids

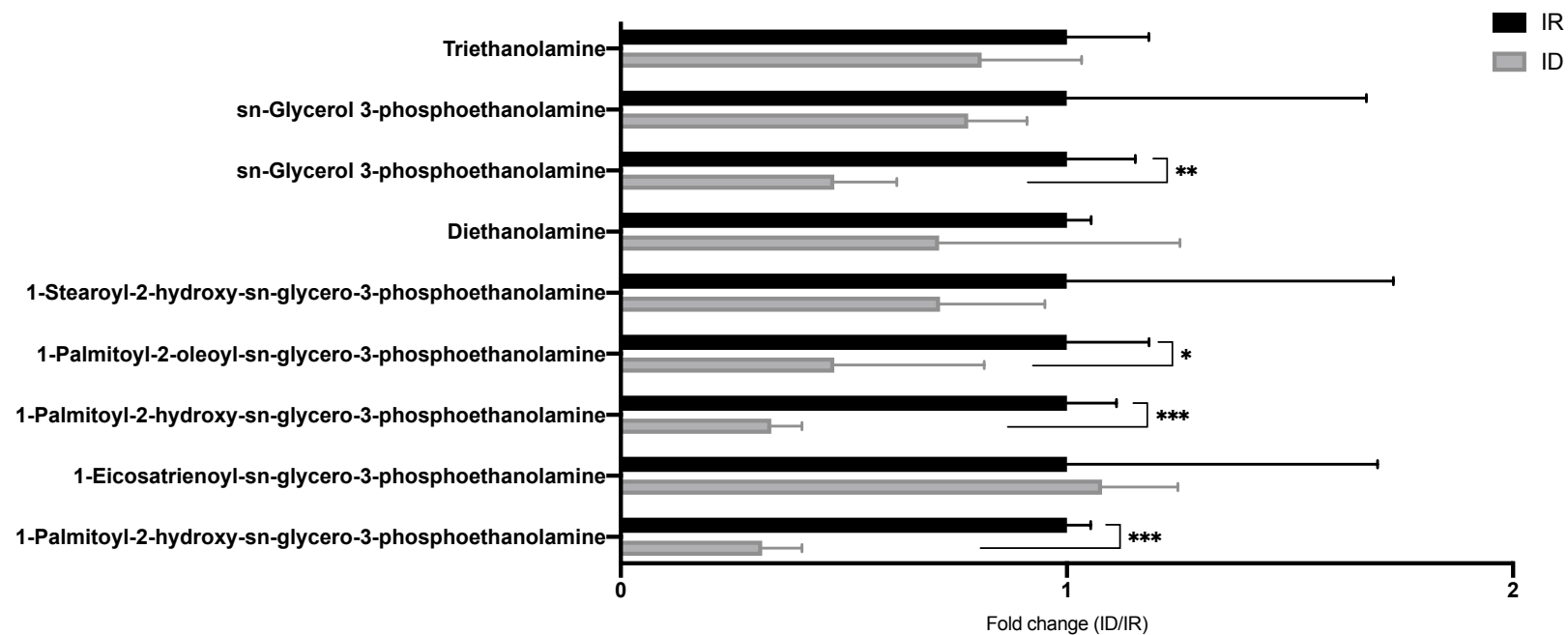

Supplement: Supplementary file 2 — Additional file 2. File 2: The relative amount of phospholipid derivatives identified in the metabolomics analysis. The relative values of phospholipid derivatives identified are shown as the fold-change from ID (gray bar) to IR (black bar) (ID/IR) cells. Significance is indicated by asterisks: *P < 0.05; **P < 0.01; ***P < 0.001. IR, iron-rich; ID, iron-deficient. [file 13071_2023_5842_MOESM2_ESM.pdf]
